# Gene Flow Complicates Phylogenetic Inference in an Archipelago Radiation

**DOI:** 10.1093/sysbio/syaf081

**Published:** 2025-11-12

**Authors:** Ethan F Gyllenhaal, Lukas B Klicka, Lucas H DeCicco, Brian C Weeks, Robert G Moyle, Michael J Andersen

**Affiliations:** Department of Biology and Museum of Southwestern Biology, University of New Mexico, 219 Yale Blvd NE, Albuquerque, NM 87131, USA; Department of Biological Sciences, Texas Tech University, 2901 Main St, Lubbock, TX 79409, USA; Department of Computational Biology, Cornell University, 350 Tower Rd, Ithaca, NY 14850, USA; Biodiversity Institute and Department of Ecology and Evolutionary Biology, University of Kansas, 1345 Jayhawk Blvd, Lawrence, KS 66045, USA; School of Arts & Sciences, Peru State College,600 Hoyt Street, Peru, NE 68421, USA; Biodiversity Institute and Department of Ecology and Evolutionary Biology, University of Kansas, 1345 Jayhawk Blvd, Lawrence, KS 66045, USA; School for Environment and Sustainability, University of Michigan, 440 Church Street, Ann Arbor, MI 48109, USA; Biodiversity Institute and Department of Ecology and Evolutionary Biology, University of Kansas, 1345 Jayhawk Blvd, Lawrence, KS 66045, USA; Department of Biology and Museum of Southwestern Biology, University of New Mexico, 219 Yale Blvd NE, Albuquerque, NM 87131, USA

**Keywords:** Archipelago, gene flow, island biogeography, Monarchidae, phylogeography, simulation

## Abstract

Allopatric divergence is a fundamental component of most traditional models of biogeography and community assembly. Gene flow between allopatric populations should be influenced by the nature of geographic barriers and can have a profound impact on adaptation, the speciation process, and phylogenetic inference. Superspecies—monophyletic groups of taxa with species-level differences in phenotype or genotype that are found exclusively in allopatry or parapatry—present an opportunity to characterize the effects of gene flow on the divergence process. Here, we investigate patterns of gene flow, population structure, and inferred phylogenetic relationships for members of an avian superspecies, the Solomons Monarchs (Aves: *Symposiachrus barbatus* complex) occupying the Solomon Islands. We found that gene flow among allopatric species matches predictions based on geography, but phylogenetic relationships were not concordant with the most likely colonization history based on a stepping-stone colonization model. Notably, the most isolated island, Makira, has a species that was inferred to be sister to the taxa on all other islands in concatenated phylogenetic analyses, despite Makira being farthest from the presumed original source of immigrants. We use population genetic simulations to demonstrate that such a result could be driven by bias resulting from low levels of gene flow, reflecting a challenge in phylogeographic inference that results when one population is differentially isolated. These simulated findings demonstrate a distinguishability issue in phylogeographic inference, where gene flow and colonization history can be difficult to disentangle.

Diversification on islands can take many forms. This includes dramatic adaptive radiation, in which speciation is associated with rapid ecological divergence and can occur despite high levels of gene flow (Seehausen 2004; Savolainen et al. 2006; Flaxman et al. 2014; Lamichhaney et al. 2018; Gillespie et al. 2020). More often, however, island taxa differentiate in allopatry and attain secondary sympatry after long periods of time, often with limited apparent functional differentiation (Rundell and Price 2009; Simões et al. 2016). These “geographic radiations” may be more stable than ecologically driven adaptive radiations, in which incomplete reproductive isolation and changing environmental conditions can lead to species collapse (Grant and Grant 2008; Rundell and Price 2009; Kleindorfer et al. 2014). The avifauna of South Pacific islands exhibit many of these geographic radiations, where secondary sympatry is relatively rare. This results in clades that display species-level phenotypic differences but have not yet attained secondary sympatry (Mayr 1942; Amadon 1966; Mayr and Diamond 2001) and have difficult-to-resolve phylogenetic relationships (e.g., McCullough et al. 2019; Brady et al. 2022). Ecological opportunity (Diamond 1977) and rapid colonization (Andersen et al. 2014), respectively, are often invoked as mechanisms driving these patterns. Although these factors undoubtedly play a role, two facets of geographic radiations liable to impact this are the frequency and drivers of gene flow between allopatric populations. Here, we explore the role gene flow may play in phylogenetic uncertainty in archipelago radiations.

Islands and island-like systems such as mountain tops and lakes offer the opportunity to better understand the population genetic processes that underly geographic radiations (Mayr and Diamond 2001; Brown et al. 2013; Manthey et al. 2020). Post-colonization gene flow among islands after a lineage colonizes an archipelago was once assumed to be rare or negligible, especially at later stages of divergence (Mayr 1942; Wilson 1961; Diamond et al. 1976). The taxon cycle hypothesis (Wilson 1961) and “great speciator” paradox (Diamond et al. 1976) posit a model in which a decline in dispersal ability occurs as species differentiate, which in turn leads to a decline in gene flow and potential for reproductive isolation. Indeed, more studies have begun to infer gene flow between distant or well-differentiated island populations (Rheindt et al. 2020; ; Mapel et al. 2021). Such continued gene flow among lineages could prohibit genetic divergence, and building an understanding of the drivers and effects of gene flow is key for understanding allopatric speciation (Slatkin 1987; Schluter 2009; Flaxman et al. 2014; Tobias et al. 2020). For example, ecology (i.e., habitat preference) predicts inter-island gene flow and within-clade species richness for bats in the Philippines (Roberts, 2006a, 2006b; Heaney and Roberts 2009).

Within groups of archipelago taxa, levels of inter-island migration are theoretically contingent on the geography of the islands they occupy. Broadly, islands are expected to experience rates of migration in accordance with island parameters such as distance from a source of colonizers, source island size, and sink target size (MacArthur and Wilson 1967; Gyllenhaal et al. 2020; Fig. 1). However, isolation can vary over time due to isostatic and eustatic sea level changes, such that some islands are separated from their neighbors by constant water gaps, whereas others that appear isolated in their modern configuration have histories of increased connectivity at periods of low sea level (Brown et al. 2013; Tan et al. 2022).

**Figure 1.**
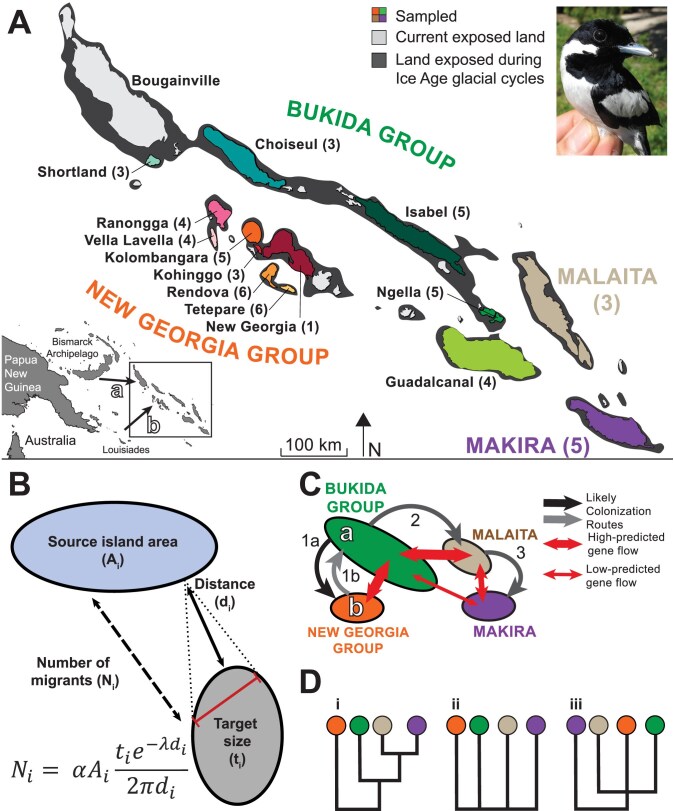
(A) Map of the Solomon Islands. Current land is colored if sampled or light gray if unsampled, and sample sizes are in parentheses after the island name. Land inferred to be exposed at the last glacial maximum is in dark gray. Inset map (from vemaps.com) shows the Solomons’ position relative to New Guinea, with hypothesized colonization routes from archipelagos in the (a) northeast and (b) southeast of New Guinea. Inset photo is of a *Symposiachrus barbatus* from Shortland Island (photo credit Mark Robbins). (B) Diagram illustrating how the number of expected migrants between islands is calculated. The equation is discussed fully in the *Materials and Methods* section: Estimation of inter-island migration (*N_i_*). Briefly, *α* represents the propensity for a source to produce migrants, *A* represents source area, *λ* represents the inverse of mean migrant over-water dispersal distance, *d* represents the distance between islands, and *t* represents the target size of the sink island. (C) Diagram illustrating two of the possible routes for colonizing the Solomon Islands and hypothetical levels of gene flow, both based on the proximity and arrangement of island grounds and common sources of colonization, namely (a) Northeast New Guinea or the Bismarck Archipelago or (b) Southeast New Guinea and the Louisiade Archipelago. Both routes are the shortest for the given starting point, based on relative proximity of island groups and the routes converge after initial colonization, with identical subsequent paths (steps 2 and 3); most importantly, regardless of the starting point, the isolated island of Makira is predicted to be colonized last. This is because Makira is relatively distant and at a disfavorable orientation (i.e., small target size) for receiving both potential colonists and therefore post-colonization migrants from the Bukida Group. Each group of islands (i.e., Bukida and New Georgia) is condensed into one ellipse representing that group. (D) Cartoon illustrations of phylogenetic patterns expected under either the route in panel C and either (i) gradual colonization (well-resolved nodes), (ii) rapid colonization (unresolved nodes), and (iii) colonization with geographically mediated migration (non-Makira branches united by gene flow).

Archipelago geography can predict both colonization history and patterns of gene flow among islands, which can impact patterns of population structure and lineage divergence. For example, connectivity of present-day islands at the Last Glacial Maximum is often a better predictor of population structure and community assembly than modern geography (Mayr and Diamond 2001; Smith and Filardi 2007; Brown et al. 2013). This led to such groups being referred to as Pleistocene Aggregate Island Complexes (PAICs; Brown and Diesmos 2002). Another common pattern is that groups that rapidly colonized archipelagoes may have short, challenging internodes (e.g., Pedersen et al. 2018; McCullough et al. 2019). Populations in such rapid radiations with high levels of gene flow may tend to be inferred to be closely related, even if the original relationships would otherwise be unresolvable via phylogenetic inference (Eckert and Carstens 2008; Leaché et al. 2014; Caujapé-Castells et al. 2017, 2022; Curto et al. 2017). Conversely, those taxa experiencing low rates of gene flow would be inferred as distantly related to other lineages in a radiation (Leaché et al. 2014; Kearns et al. 2018). This disparity between high and low levels of gene flow could bias phylogenetic inference in a given archipelago, such that taxa on the most isolated islands are inferred as sister to less isolated taxa. This can in turn impact biogeographic inferences that leverage topologies to infer ancestral range transitions and character inference (e.g., Omland 1999; Matzke 2014). Therefore, understanding drivers of gene flow within archipelagos is critical to understanding potential biases in phylogenetic inference, and in turn the distinguishability of alternate scenarios of colonization and gene flow. However, few studies have attempted to develop a theoretical understanding of these biases and how they affect inferred topologies in a geographic context (but see Leaché et al. 2014).

The Solomon Islands are an oceanic archipelago in the Southwest Pacific (Fig. 1A) that contains several co-distributed clades of birds across its main islands (Mayr and Diamond 2001; Smith and Filardi 2007). The distribution of taxa across the Solomon Islands is largely concordant with the modern islands that once formed PAICs during the Last Glacial Maximum when sea levels dropped by approximately 134 m (Fig. 1; Heaney 1986; Lambeck et al. 2014; Tozer et al. 2019; https://www.ncei.noaa.gov/maps/bathymetry/). The Bukida Group PAIC (Bukida Group hereafter) comprises the main northern chain of islands, from Bougainville to Guadalcanal, and when connected is referred to as Greater Bukida (Fig. 1A). The New Georgia Group islands consist of multiple PAICs, and all islands in the group have always been isolated from the Bukida Group. As such, many taxa are shared among islands in the New Georgia Group that are distinct from those in the Bukida Group. Similarly, the island of Malaita has remained isolated and harbors distinct taxa, although potentially fewer than expected given its size and geographic isolation (Mayr and Diamond 2001). Makira is the easternmost major island in the archipelago and is the farthest from any other major island. Makira’s relative isolation has resulted in globally significant levels of avian endemism, notably higher than nearby Malaita or the New Georgia Group (Mayr and Diamond 2001).

The relatively linear geographic arrangement of the major islands within the Solomon Archipelago facilitates exploration of gene flow across multiple scales in an island biogeographic framework. First, we can predict that divergence will be higher between PAICs than within PAICs, even when PAICs are in close proximity such as those within the New Georgia Group. Second, we can predict that phylogenetic inference may be shaped by the relative proximity of the island groups. Because many taxa colonized the archipelago from the west (e.g., from New Guinea and the Bismarck Archipelago; Mayr and Diamond 2001), the relative proximity of island groups and island biogeographic theory lead to a prediction that Makira was likely colonized last given its placement at the eastern end of the archipelago (most parsimonious routes in Fig. 1C). Therefore, if colonization timing alone determines patterns of phylogenetic divergence, taxa on Makira should be nested within clades of the other groups (Fig. 1Di). If colonization was rapid and followed by immediate cessation of gene flow, a hard polytomy at the root of the Solomon Islands clade would be produced (Fig. 1Dii). Malaita and the New Georgia Group are relatively close to the Bukida Group in terms of geographic distance and have a favorable orientation for exchanging migrants (i.e., running parallel to the Bukida Group). Makira is farther from the Bukida Group and is a small target for migrants. Therefore, if gene flow is sufficient to genetically connect Malaita and the New Georgia Group to the Bukida Group, we would expect lineages on Makira to be sister to all other lineages in the archipelago, despite those lineages presumably colonizing Makira after having colonized the other islands in the archipelago (Fig. 1Diii). This pattern of Makira being inferred as sister to other island groups has been shown in several data sets (e.g., Klicka et al. 2022; Lavery et al. 2023, DeRaad et al. 2024), with some analyzing only small genetic regions (Pulvers and Colgan 2007; Hagen et al. 2012; Andersen et al. 2014; Jønsson et al. 2014) or lacking a strongly supported topology (McCullough et al. 2019).

Superspecies in the Solomon Islands are a model for investigating dynamics of geographic radiations. The Solomons Monarch (*Symposiachrus barbatus*) complex is distributed across the main Solomon Archipelago, from Bougainville to Makira, where three to four allopatric species are currently recognized (Dickinson and Christidis 2014; Gill et al. 2022). Of these, *S. barbatus* is the most widespread species and is distributed across the Bukida Group. *Symposiachrus malaitae* is restricted to Malaita and is treated variably as a separate species or a subspecies of *S. barbatus. Symposiachrus browni* is endemic to the New Georgia Group, where it is composed of four subspecies. Finally, *S. vidua* of Makira is consistently regarded as a distinct species.

Here, we use two approaches to test the hypothesis that historical gene flow that is concordant with island geography shapes phylogeography. First, we test whether population structure is concordant with PAICs and whether the phylogeny reflects a likely colonization route with genomic data from the Solomons Monarch complex. Second, we test our prediction that gene flow can drive this pattern using population genetic simulations based on theoretical levels of gene flow derived from the islands occupied by the Solomons Monarchs. In doing so, we test the role of island arrangement and history of connectivity in shaping inferred patterns of divergence and characterize the conditions under which differential isolation may bias phylogenetic.

## Materials and Methods

### Sampling Scheme

We sampled 64 individuals for our focal phylogeographic analyses, of which 57 comprised our ingroup of the Solomons Monarch complex, and the remainder represented four outgroup taxa included to root phylogenies and to determine the ancestral allele for ABBA/BABA tests (one *Symposiachrus guttula*, one *S. infelix*, two *S. trivirgatus*, and three *S. verticalis*). All samples were from specimen-vouchered tissues housed at three natural history collections (Supplementary Table S1). For the Solomon Islands samples, 30 were from the New Georgia Group, 21 from the Bukida Group, three from Malaita, and five from Makira (Fig. 1A). Our sampling included all focal species (*Symposiachrus barbatus, S. browni, S. malaitae*, and *S. vidua*) and subspecies in the *S. barbatus* complex from all relevant islands and island complexes, except for Bougainville. Samples from Shortland Island (connected to Bougainville during Pleistocene eustasy) served as a substitute.

### DNA Extraction, Library Prep, and Sequencing

We extracted genomic DNA using a manual magnetic bead-based protocol (https://github.com/phyletica/lab-protocols/blob/master/extraction-spri.md) based on Rohland and Reich (2012), and eluted DNA from beads using 1X TE buffer. All DNA extracts were standardized to 5 ng/μL of DNA and plated at 10 μL per sample (50 ng of DNA). Standard MSG (Multiplexed Shotgun Genotyping) RADseq libraries (Andolfatto et al. 2011) were prepared at the KU Genome Sequencing Core facility. Briefly, genomic DNA was digested using the enzyme NdeI, bar-coded adapters were ligated to samples to allow for multiplexing, DNA fragments were size-selected for 495–605 bp range, size-selected fragments were amplified using PCR, and amplified samples were cleaned using AMPure beads to remove primer dimers and short-fragment DNA. Single-read 100-bp Illumina sequencing was performed on a high output run of a NextSeq 550 machine.

To better assess the origin of colonization in the Solomon Islands, we also inferred a phylogeny based on ultraconserved elements (UCEs; Faircloth et al. 2012). This approach leveraged data from plus additional unpublished data that we produced and included eight ingroup samples (two per island group) and 17 outgroup samples (10 distinct populations; Supplementary Table S2). See for a detailed description of the procedures we followed. In short, these samples were derived from genomic libraries enriched for thousands of conserved regions across the genomes.

### Bioinformatics

We followed the Stacks v2.41 (Rochette et al. 2019) reference-based assembly pipeline to call Single Nucleotide Polymorphisms (SNPs) from our RAD-seq data set. The *process_radtags* module in single-end mode was used to demultiplex our reads, and we removed samples with fewer than 300,000 reads. These reads were then aligned to a *Corvus moneduloides* reference genome (RefSeq GCF_009651085.1; Dussex et al. 2021) using the *mem* algorithm of BWA v0.7.17 (Li and Durbin 2009). This was the closest chromosome-level reference available to us at the time of analysis, sharing a common ancestor with our study taxon approximately 17 million years ago (McCullough et al. 2022) and resulting in us successfully mapping 86% of reads. The resulting SAM files were sorted and converted to BAM files using SAMtools v1.10 (Li et al. 2009). Finally, the Stacks programs *gstacks* and *populations* were used to generate several data sets for different analyses. Relevant subsets are described in Supplementary Table S1. Resulting output files were used for downstream analyses. Unless otherwise stated, the analyses were performed using a 75% complete matrix of biallelic SNPs and we included only one random SNP per locus to account for linkage disequilibrium. All filtering was performed in Stacks v2.41. The results presented are comparable with exploratory de novo assembly (not presented).

For the UCE data set, we produced concatenated input files using phyluce v1.7.1 (Faircloth 2015). We did not have a strict read cutoff as with the RADseq, as all samples were known to have sufficient coverage prior to this study (lowest sample had 1.4 million reads). We assembled contigs using SPAdes v3.14.1 (Bankevich et al. 2012), aligned locus-specific data sets with mafft v7.475 (Katoh and Standley 2013), and internally trimmed those data sets with gblocks v0.91b, changing only the b2 parameter to 65% (Castresana 2000).

### Population Structure Analyses

We performed two population structure analyses for all populations of our ingroup. The first analysis was the calculation of Weir and Cockerham’s (1984) F_ST_ from SNPs between each island population using a custom wrapper around vcftools v0.1.15 (Danecek et al. 2011; wrapper first developed for Mapel et al. 2021; updated script can be found at https://github.com/ethangyllenhaal/SolomonsSymposRad). The second analysis assessed genetic clustering using principal component analyses (PCA) from the SNP data using adegenet v2.1.1 (Jombart 2008) in R v3.6.1 (R Core Team 2019), with inputs produced from the R package vcfR v1.8.0 (Knaus and Grünwald 2017). This analysis was performed hierarchically (i.e., between and within island groups) to assess the impact of PAIC connectivity on population differentiation. Clustering was first for all the Solomon Islands, then for both the Bukida and New Georgia groups. See Supplementary Material (“Assessing a putative intergrade”) for methods on a putative F1 inter-island intergrade.

### Phylogenetic Analysis

We used three methods for generating phylogenetic trees for RAD-seq data and two methods for UCE data. The shared methods were sequence-based (rather than variant-based) trees. For RAD-seq data, we generated alignments using the Stacks *populations* module’s “phylip-var-all” parameter to produce an alignment with all variant and invariant sites in the data set, with a 75% complete matrix of loci, in which at least 75% of the samples had data for a particular locus to be included. For UCE data, we made alignments with a 90% complete matrix due to higher matrix occupancy of the data set. The first phylogenetic method was an unpartitioned concatenated maximum likelihood approach with IQTREE v2.0.3 (Minh et al. 2020) with 1000 ultrarapid bootstraps (Hoang et al. 2018) and automated model selection performed by ModelFinder (Kalyaanamoorthy et al. 2017). Second, we used these same data sets with locus partitions to generate input for SVDQuartets in PAUP* v4.0a169 with 100 bootstraps and evaluating all quartets (Chifman and Kubatko 2014). We grouped samples from different islands using taxon partitions for this analysis. We generated input for out final analysis with a script from https://github.com/BEAST2-Dev/SNAPP/tree/master/script and custom scripts (found at https://github.com/ethangyllenhaal/SolomonsSymposRad) to generate input files for SNAPP v1.5.1 (Bryant et al. 2012). This used a 100% complete VCF with a given subset of samples (see below) as input, with each VCF output using the Stacks *populations* module. We ran our focal SNAPP analysis in BEAST v2.6.3 (Bouckaert et al. 2019) for 10 million generations, discarding 1 million generations as burn-in and used three random individuals (i.e., lowest island-group-specific sample size) from a given island per island group, with *S. trivirgatus* (the most closely related taxon in the data set; Andersen, Hosner, et al. 2015) as an outgroup. We used mutation priors automatically calculated in BEAUti but otherwise used default priors. Two additional analyses were performed to assess consistency of the SNAPP results by expanding sampling within the Bukida and New Georgia groups: one with two individuals from two islands per group and one with two individuals from five islands per group. We assessed convergence using Tracer v1.7.1 (Rambaut et al. 2018), focusing on Effective Sample Size (ESS)  ≥ 200 in all parameters other than thetas and confirmed convergence using the consistency in the tree topology in the posterior as additional generations ran. Trees from the posterior after the burn-in were combined and visualized in DensiTree v2.2.7 (Bouckaert 2010) and TreeAnnotator v2.6.0 (Bouckaert et al. 2019).

### Assessment of Gene Tree Support

To precisely characterize how gene tree heterogeneity manifested itself in this data set, we performed two methods. First, we evaluated the characteristics of gene trees supporting specific topologies (constructed in IQTREE v2.0.3; Minh et al. 2020) for loci with at least two parsimony-informative sites (as assessed by phykit v 1.13.1; Steenwyk et al. 2021). Topology and phylogenetic distance were then inferred using custom python script using ETE3 v3.1.3 (Huerta-Cepas et al. 2016) to work with the input trees. The first metric estimated was the frequencies of focal topologies, primarily what island group’s population was sister to the rest. For those cases, the mean phylogenetic distance between the sister taxon and the other three taxa was estimated as well.

### Tests for Gene Flow

We tested for gene flow with ABBA/BABA tests as implemented in Dsuite v0.4r38 (Malinsky et al. 2021), using samples of *S. trivirgatus* as the outgroup. We calculated the D statistic (Durand et al. 2011) and its significance in addition to the f_4_ (Patterson et al. 2012) estimate of admixture proportion. We focused on specific comparisons among different island groups. We excluded any population represented by only one sample, an admixed individual from Ranongga, and six older degraded samples. The individual from Ranongga was excluded to maintain consistent phenotypic representation and avoid biasing estimates of gene flow from a presumed recent migrant or intergrade. We tested multiple topologies and sampling schemes, including the exclusion of certain populations to ensure robustness to topological uncertainty. Examples of such data sets include ones that excluded Malaita and New Georgia. We also randomly downsampled our data sets to two and three individuals per population to further ensure that uneven sampling was not biasing the results. We focus the discussion of our results on gene flow that was consistently inferred across all relevant analyses. To account for the partial independence of tests, we used the Benjamini–Hochberg procedure (Benjamini and Hochberg 1995) to provide a false discovery rate for tests in each category of edge (i.e., Bukida to Malaita, within the New Georgia Group). We also calculated the harmonic mean *P*-value for the same categories (Wilson 2019). We also used the f_branch_ function to make inferences about timing of gene flow (Malinsky et al. 2018).

### Colonization Simulations

We propose a probable route of colonization in Figure 1, but its importance to our hypothesis and future simulations require a more thorough test. As such, we developed a simulation framework to determine the theoretical frequency of different colonization routes through the Solomon Islands. To do this, we used the spatial mode of the forward population genetic simulator SLiM 4 (Haller and Messer 2023) and raster files generated in PleistoDist (Bintanja and Van De Wal 2008; Tan et al. 2022). Full simulations are described in Supplementary Methods.

### Estimation of Inter-Island Migration

To generate expectations of inter-island gene flow, we used estimates of inter-island migration rates based on a framework derived from MacArthur and Wilson (1963 and 1967), described in Gyllenhaal et al. (2020), and summarized in Figure 1B. These estimates are the product of the number of potential migrants, the probability a random migrant will go toward the destination island, and a dispersal kernel to model failed dispersal (measured in kilometers). The number of potential migrants represents the individuals in each generation that will attempt to cross the oceanic barrier and potentially immigrate to new islands. It is determined by island area multiplied by the propensity for that area to produce migrants (α) or, for the simulation, by the population size and the propensity for individuals to migrate. Assuming these migrants randomly choose a direction to fly, the probability that a random migrant will go toward the target island is the proportion of angles (out of a 360^o^ circle) from which a straight line can be drawn from the source to the target. For our purposes, it is determined by the size of the target island relative to the source and the distance to the source. Finally, the dispersal kernel is a mathematical relationship between distance traveled and probability of success for an individual dispersing that distance. Here, we use an exponential distribution with a variety of mean dispersal distances (following MacArthur and Wilson 1967). The product of the probability a migrant will go toward the target and the distance-decay equation is the probability a given migrant will reach the target island. The inter-island distances and target size were primarily estimated in PleistoDist v1.1.0 (Tan et al. 2022), supplemented by Google Maps (2024) for certain cases, such as estimating the target size of the geographically complex New Georgia Group. It is worth noting that ratios of two unidirectional migration edges eliminate the challenge of estimating propensity to produce migrants (α), and we refer to this ratio in the *Discussion* section.

### Phylogeographic Simulations

Even with a known colonization route, our hypothesis rests on the claim that island-mediated gene flow alone can obscure inferences of evolutionary history, in particular demonstrating that biased inference can theoretically occur in the Solomon Islands. To see how gene flow shapes inference, we developed a backward simulation framework in msprime v1.0.1 (Baumdicker et al. 2022). We simulated five populations corresponding to the four island groups and a source population (used as an outgroup). The cumulative population size of all ingroup island groups was set to a single value, with proportional share of individuals for each island group determined using the number of individuals in spatial simulation akin to the ones described above after all islands have been colonized for a stable number of generations. Migration between populations was determined as described in the *Estimation of Inter-Island Migration* section (see Fig. 1B), using a proportion of potential migrants of 5 × 10^−5^ (based on exploratory analyses), target size and distance estimated primarily in PleistoDist (Tan et al. 2022), and allowing mean dispersal to vary between simulations. Source population sizes were based on the method described above for groups other than when Bukida was the source, where the proportion was based on the closest large islands (i.e., Choiseul and Isabel for the New Georgia Group, Guadalcanal and Isabel for Malaita, and Guadalcanal and Makira). Going forward in time, splitting events occurred as follows (matching route b in Fig. 1C): an ancestral population of 10,000 individuals split into an outgroup (*n* = 10,000), then a Bukida Group population, which served first as a source for the New Georgia Group population, then the Malaita population, and finally the Malaita population was the source for the Makira population, followed by a longer period of evolution. Migration occurred throughout, and split events occurred with a “trunk model” where the source population persisted unaffected by the divergence. We explored this parameter space for combinations of total effective population size (50k, 100k, and 200k), post-split divergence time (200k, 400k, 800k, 1.6M, and 3.2M generations), and time between colonization events (25k, 50k, 100k, 200k, and 400k generations). To demonstrate the generality of gene flow disrupting phylogenetic inference, we also performed a set of simulations (for the main parameters described above) with Makira as the first island colonized, followed sequentially by Malaita, Bukida, and New Georgia populations. We added mutations to the resulting tree sequences using msprime’s sim_mutations method under a Jukes–Cantor (JC) model and a per-generation mutation rate of 2.3 × 10^−9^ (Smeds et al. 2016), output fastas using tskit v0.5.2 (Kelleher et al. 2018), and reduced the matrix to only variant sites using a custom script (https://github.com/ethangyllenhaal/SolomonsSymposRad). The resulting output from each simulation was used as an input for IQTREE v2.0.3 (Minh et al. 2020), which we ran with 1000 ultrafast bootstraps (Hoang et al. 2018) and a JC model of sequence evolution (i.e., the same model used to generate mutations). We ran 50 replicates in GNU Parallel (Tange 2021) for each parameter combination. Topologies of the output trees were automatically assessed using a custom python script using ETE3 v3.1.3’s (Huerta-Cepas et al. 2016).

## Results

### Sequencing Information

Of the 67 sampled individuals, we removed two due to an insufficient number of reads and one due to apparent siblingship due to excess clustering in PCAs. The 64 individuals that remained are listed in Supplementary Table S1. The effective per-sample coverage (mean number of reads per locus for a given sample) had a mean of 89.5x (range of 22.2–334.7x). The mean number of total (variant and invariant) sites per locus in the full RAD-seq data set was 96.4. The 75% complete data set of all individuals resulted in a total of 15,340 loci and 1,479,462 sites, of which 51,177 were variant (mean of 3.4 variable sites per locus). Other data sets (with members in Supplementary Table S1) ranged from 10,484 to 15,261 loci, depending on taxa included and completeness of the matrix. The UCE data set averaged 3.2 million clean reads per sample for the full data set, and 2.1 million for samples not in . Our concatenated, 90% complete UCE data set contained 4096 loci averaging 767 bp in length (3.14 Mb in total), with a mean of 5.72 informative sites per locus (23,433 total).

### Population Structure

**Figure 2.**
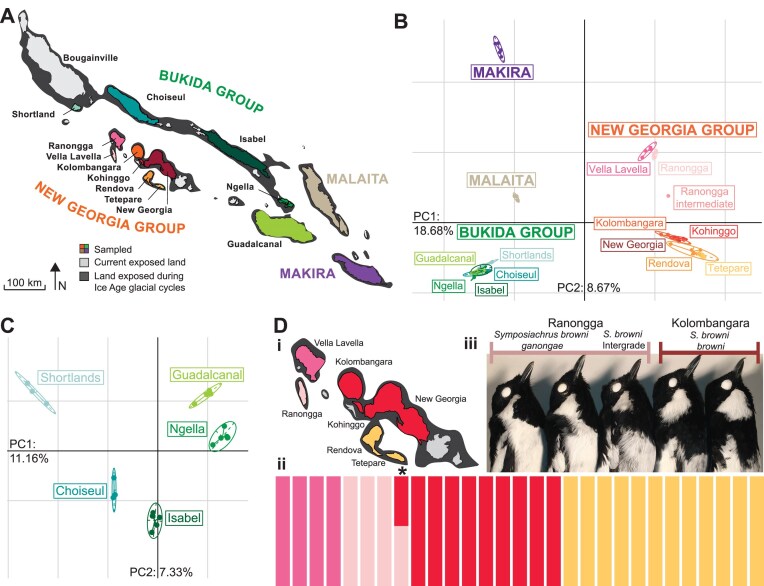
(A) Map of the Solomon Islands, colored to match B and C. (B) Principal component analysis (PCA) of all sampled individuals, labeled by island (other than Ranongga intermediate, see (D). (C) PCA of all Bukida Group samples. (D) Admixture analysis of all New Georgia Group samples, with (i) the map of the New Georgia Group colored according to grouped islands in (ii) the admixture plot of the samples, with the putative intergrade marked with an asterisk. (iii) Representative specimens from the Museum of Southwestern Biology from Ranongga (left two) and Kolombangra (right two), with the Ranongga intermediate in the middle. The intermediate Ranongga individual is the only notably admixed sample.

We found that most island populations (hereafter referred to by their island names) formed distinct genetic clusters in PCAs. Islands that were connected at glacial maxima were only differentiable in PCAs that were specific to their respective island groups (Fig. 2B–C; Supplementary Fig. S1). The only exception to this pattern was the islands of New Georgia, Kohinggo, and Kolombangara, which were largely indistinguishable in any PCA (Supplementary Fig. S1b–c). Such connected island groups also had lower pairwise F_ST_ (Supplementary Table S3). In the Bukida Group, we found that large islands that were previously connected during Last Glacial Maximum (LGM) were the least differentiated (e.g., Choiseul and Isabel F_ST_ = 0.036; Supplementary Table S3). We observed a pattern of PCA proximity concordant with the linear arrangement of current islands in the Bukida Group, with gaps between different populations (Supplementary Table S3; Fig. 2C). Additionally, we found that populations on small islands had elevated F_ST_, consistent with the expectation of a more rapid increase of F_ST_ over time due to drift in small populations (Holsinger and Weir 2009). Admixture (Alexander and Lange 2011) analyses revealed that an unusual individual on Ranongga (voucher MSB: Birds:55053) was equally admixed between *S. b. ganongae* and *S. b. browni* (Fig. 2D). Its high heterozygosity of SNPs fixed between parental populations (0.878) confirmed it is likely an F1 intergrade (methods in Supplementary Material, “Assessing a putative intergrade”).

### Phylogenetic Analysis

**Figure 3.**
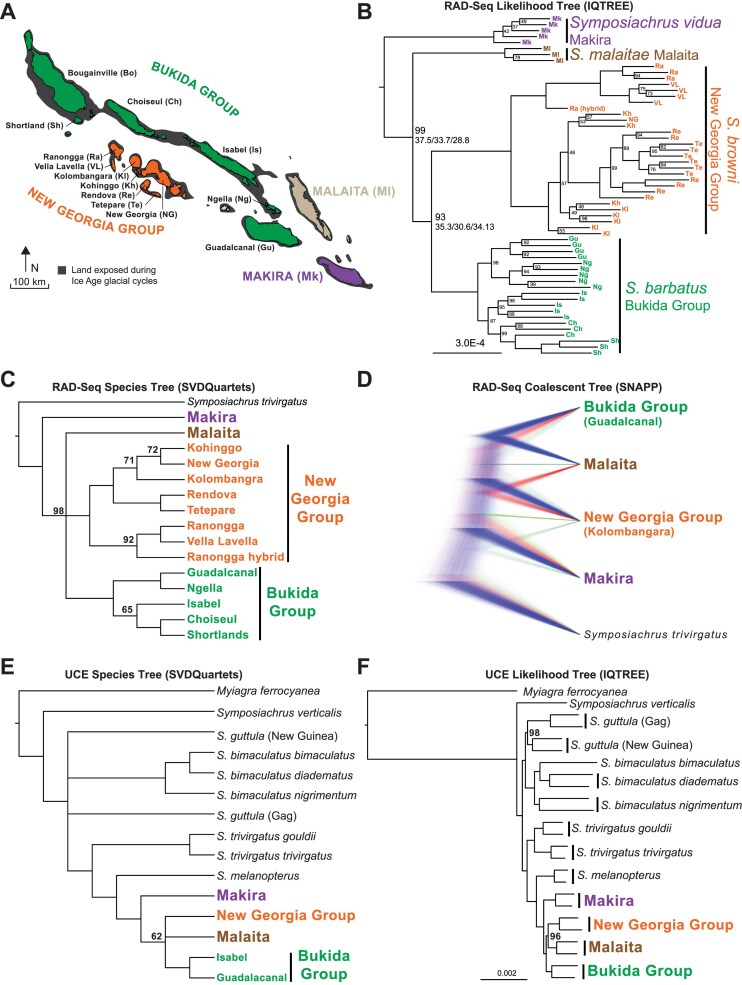
Phylogenies of focal Solomon Island taxa with an outgroup of *Symposiachrus trivirgatus*. Bootstrap values below 100 are displayed on the nodes, and nodes with less than 60% support are collapsed. Outgroups are listed in black italic text. (A) Map of the Solomon Islands, with island names and letters corresponding to focal populations. Colors correspond to island group. (B) Concatenated maximum likelihood phylogeny of RAD-seq data of all Solomons samples generated in IQTREE, note the uncertainty within Pleistocene island complexes. Focal nodes for the relationships among the four island groups also have the site concordance factor and two site discordance factors (as percentages). Tips are labeled according to group color with letters corresponding to island group. Outgroups are not shown here, see Supplementary Fig. S2. (C) Cladogram of SVDQuartets species tree of RAD-seq data, with samples grouped by island. (D) SNAPP tree of RAD-seq data of focal islands from each of the four focal island groups, with island names in parentheses below group names when relevant (see Supplementary Fig. S3 for different sampling schemes). Note that no nodes within the Solomons are well-supported (poster probability < 0.95). (E) SVDQuartets cladogram of UCE data, with samples grouped by island. (F) IQTREE phylogeny of UCE data, with bars over tips representing members of a given taxon or island group.

Most of our phylogenetic analyses resulted in patterns where the taxon on the isolated island of Makira was sister to other taxa in the Solomon Islands, but support for this inference varied greatly from well-supported to negligible. In maximum likelihood analyses of the RAD-seq data set, Makira was inferred to be sister to other islands in the archipelago with high but imperfect bootstrap support (99). This IQTREE analysis also inferred the Bukida Group was supported as sister to the New Georgia Group with moderate bootstrap support (93), and these were in turn sister to Malaita (Fig. 3B). Species tree analyses often recovered a similar topology but lacked the support of the concatenated analysis. Our SVDQuartets analysis of the RAD-seq data set found Makira sister to the other three populations but was otherwise equivocal (Fig. 3C). Of our SNAPP analyses, only one recovered a well-supported relationship (posterior probability > 0.95) among the island groups in the Solomons, which had two individuals per island group. Other analyses were not well resolved (including Fig. 3D; Posterior probability = 0.90). The IQTREE inference on the UCE data set inferred high support for a Makira-sister relationship, but a different topology from other analyses (Malaita and New Georgia as sister, and Bukida sister to those; Fig. 3F). Results from SVDQuartets inference with UCE data failed to resolve relationships within the Solomons (bootstrap support = 62; Fig. 3). One finding from both UCE analyses key to assessing the start point of colonization was that the *S. barbatus* complex was sister to *S. melanopterus* (formerly *S. trivirgatus melanopterus*; McCullough et al. 2021), and that clade was sister to *S. trivirgatus*. This novelty is due to the increased sampling in this data set relative to the RAD-seq data set and past work on the genus.

### Assessment of Gene Tree Support

For our data set filtered to four ingroup and one outgroup, 224 loci had two or more parsimony-informative sites and were used for analyses of gene trees (Supplementary Table S4). Other those gene trees, 28% found Makira to be sister, followed by 18% New Georgia, 17% Bukida, and 12% Malaita (25% of gene trees did not fit into this category). For the subset with a single taxon inferred as sister to the rest, the branch length between the sister taxon and the other three was lowest for the Makira-sister trees, followed by New Georgia sister, Malaita sister, and Bukida sister. These metrics were similar for subsets with three and one parsimony informative sites as well (Supplementary Table S4).

### Tests for Gene Flow

**Figure 4.**
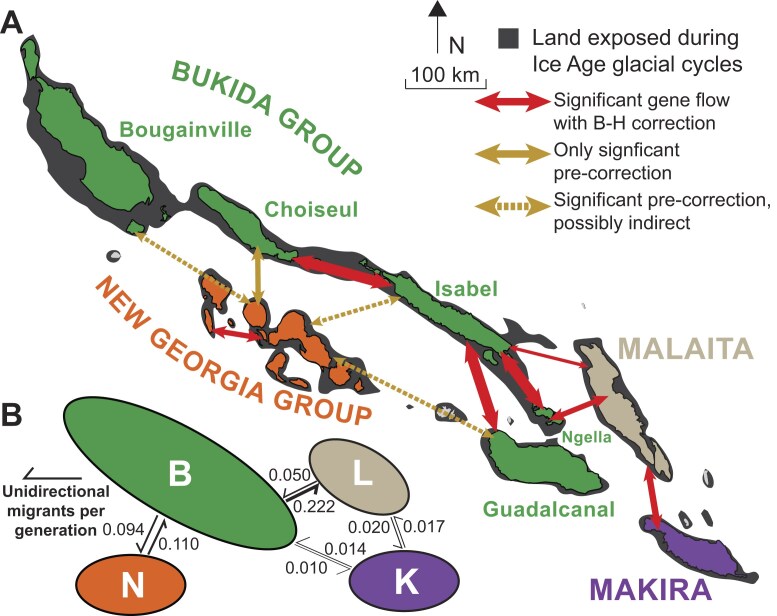
(A) Map of the Solomon Islands overlayed with statistically significant gene flow inferred by DSuite. Width of arrows relates to the inferred admixture proportions (f_4_ statistic). Finely dashed arrows represent edges not inferred with the fbranch statistic, presumably due to gene flow between Choiseul and other members of the Bukida Group. Coarsely dashed arrows represent gene flow assumed to occur between sister populations in the Bukida Group. (B) Levels of inter-island-group gene flow (in units of number of migrants per generation) based on island biogeographic theory, as used in phylogeographic simulations for a mean dispersal distance of 35 km.

We found evidence for gene flow across the *S. barbatus* complex (Fig. 4A; Supplementary Fig. S4). Five of these patterns were consistent across multiple individual tests and combinations of populations, and four recovered both positive results after false discovery rate (FDR) corrections and had harmonic *P*-values less than 0.05 (Supplementary Table S5). First, we found evidence for high levels of gene flow (f_4_ > 0.15) between adjacent islands in the Bukida Group when it was possible to test (harmonic *P* < 0.001). Second, we found evidence for gene flow between islands of the main New Georgia Group (Kolombangara, Tetepare, and Rendova) and Ranongga, although only gene flow between Tetepare and Ranongga was significant after FDR correction (harmonic *P*-value within New Georgia = 0.024). Curiously, we found no evidence to suggest gene flow between Vella Lavella and other islands in the New Georgia Group, despite their large land area size and close physical proximity. Third, we found evidence for gene flow between Malaita and the Bukida Group (harmonic *P* < 0.01). Fourth, we found evidence in almost every possible comparison of gene flow between Malaita and Makira (Supplementary Table S5; harmonic *P* < 0.01). We found weak support for gene flow between Malaita and Vella Lavella, but we caution against overinterpretation of this result because it occurred in only one comparison (Supplementary Table S5). Finally, we found some evidence for gene flow between all islands of the Bukida Group and the main New Georgia Group. However, it did not hold up to FDR correction and had a harmonic *P*-value of 0.059. The f_branch_ statistic suggested the gene flow was with Choiseul and an ancestor of current New Georgia populations (Supplementary Fig. S4).

### Colonization Simulations

**Figure 5.**
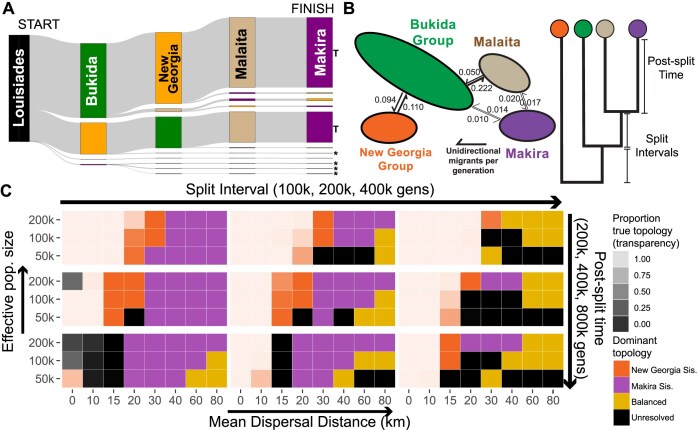
Summary of results of our forward (A) and backward (B–C) population genetic simulations. (A) Sankey diagram illustrating colonization order, summed across dispersal values (which displayed similar values). Note that this corresponds to the order in which islands were occupied, not an exact colonization history, gray connections are for illustrative value. Paths matching the “true” topology (as used in backward simulations) are marked with a “T,” whereas those matching a Makira-sister pattern are marked with a “*.” (B) A simplified map showing values of gene flow used for simulations with a 35 km mean dispersal distance and illustration of the input topology and meaning of parameter values for backward simulations. (C) Results of backward simulations. The color of each square corresponds to the most frequent topology, and transparency corresponds to the proportion of simulations that recovered the true topology in panel B. For visualization purposes, the dominant topology was first assigned as unresolved if less than 50% of the trees found a well-supported topology of monophyletic island groups, and if not, the dominant topology represents the majority of the remaining, well-resolved topologies. The inner axis labels correspond to variation in effective population size and dispersal within each set of tiles, whereas the outer labels correspond to values of split times that are fixed for a given set of tiles.

Several consistent patterns emerged from our colonization simulations and were largely similar across mean dispersal values (Fig. 5A; Supplementary Table S6). The island group first colonized was most frequently the Bukida Group (58–75% of successful simulations where colonization occurred), with most other first colonization events in the New Georgia Group (25–34% of successful simulations). A small subset found other islands were colonized first, generally only representing 1% of the simulations for a given dispersal distance. The exception was for 400 pixels, where Makira was colonized first in 8% of successful simulations, but only 24 simulations managed to achieve colonization at that value. Within these categorizations, colonization order was largely consistent, with Makira most often being colonized last, and Malaita often second-to-last. Finally, these simulations rarely recovered a scenario that could result in a topology in which Makira would be sister to other islands (1% across all simulations), whereas 94% produced a pattern where New Georgia was sister. Additionally, we found that with these dispersal values, Makira often had a high level of mixed founding populations between Bukida and Malaita.

### Phylogeographic Simulations

We simulated the effect of geography-mediated gene flow on phylogenetic inference and found that when the true topology was recovered, it was obscured by even low levels of gene flow for both colonization directions (Fig. 5C; Supplementary Fig. S5). However, when shorter time intervals were used between splits within the Solomon Islands, there was often insufficient resolution to produce a supported topology at low gene flow, and resolved topologies were only recovered at moderate levels of gene flow (Supplementary Fig. S5). All dispersal values correspond to a relatively low level of gene flow, with a mean dispersal distance of 40 km corresponding to less than one migrant (0.898) per generation simulated across the entire archipelago. The most impactful variable for ability to infer the true topology was the amount of time between colonization steps (Fig. 5C), with longer post-divergence evolutionary time resulting in long branches and poorly resolved topologies (Supplementary Fig. S5). When intermediate levels of gene flow were permitted, Makira-sister topologies dominated in many (but not all) parameter combinations. Curiously, the highest levels of gene flow tended to produce a balanced topology, particularly the one with Makira and Malaita sister. For simulations where Makira was truly sister to other populations, we found that the true topology was only observed at low levels of gene flow, with an alternate topology (the same one found in most of the previously described simulations) emerging as dominant and intermediate dispersal values.

## Discussion

We find that empirical populations across an oceanic archipelago experience levels of gene flow consistent with past and present island geography (Figs. 2 and 4). We also used population genetic simulations to demonstrate that low levels of gene flow based on this island geography can bias phylogenetic inference (Fig. 5). Additionally, although several inference methods were inconclusive, all well-resolved phylogenetic analyses inferred the island of Makira as being sister to all other islands (Fig. 3), which could suggest that it was the first island in the Solomon Islands to be colonized by *Symposiachrus*. However, this scenario seems improbable given two aspects of the system: (1) Makira is the easternmost island in a largely linear archipelago, and *Symposiachrus* taxa are not found east of the Solomon Islands, and (2) the islands of the New Georgia Group are inferred to be the most-likely point of colonization based on the location of the sister taxon to the Solomon Islands *S. barbatus* complex (Fig. 3E–F; see the *Biogeography of Symposiachrus* section). Our colonization simulations suggest that it is unlikely that Makira was the first or second island to be colonized, which could produce a topology in which it is sister to other islands (Fig. 5A). However, taken at face value, our phylogenetic data suggest it could be the first island colonized. Therefore, we used population genetic simulations to demonstrate that low levels of gene flow (i.e., less than one migrant per generation archipelago-wide, Fig. 5B) can consistently result in the inference of topologies in which Makira is sister to other islands (Fig. 5C), bringing interpretations about colonization history into question. This potential bias in phylogenetic inference driven by gene flow is especially visible in archipelagos and warrants a degree of caution with phylogenetic inference in the face of heterogenous gene flow. We found this signal most strongly for concatenated analyses but note that coalescent relationships were equivocal for our empirical data. If geographic radiations such as those in archipelagos are important for generating biological diversity at deeper time scales (Rundell and Price 2009; Simões et al. 2016), our findings suggest nodes stemming from such radiations may require caution when interpreting them as indicative of colonization order, as colonization histories are not necessarily distinguishable from phylogeographic data.

### Simulated Effects of Intra-Archipelagic Dispersal

We performed two sets of simulations to better evaluate the support for the theoretically most-likely colonization order and to demonstrate the theoretical impact that geography-correlated gene flow can have on phylogenetic inference. Our colonization simulations, with a starting point based on the current range of the sister species to the *Symposiachrus* of the Solomon Islands, suggest that it was most likely that Makira was the last island to be colonized (Fig. 5A); 94% of colonization routes produced a topology with the New Georgia Group sister to all other islands in the archipelago (Fig. 5A). However, colonization is not always linear (e.g., Cibois et al. 2011), and we deemed it prudent to demonstrate the feasibility of geographically mediated gene flow to produce the Makira-sister pattern more generally.

To test our prediction that low levels of inter-island gene flow alone could produce the Makira-sister topology, we conducted a series of population genetic simulations. We found that when the input topology was able to be recovered in scenarios without gene flow, low levels were sufficient to erase that signal, even when the time between colonization events was long (Fig. 5C). Our focus is on the simulated history that is most likely, given theoretical expectations (i.e., Makira colonized last). However, even for simulations where Makira was colonized first, gene flow resulted in the inference of an alternate Makira-sister pattern wide range of dispersal values (Supplementary Fig. S5b). Phylogenetic inference from our Bukida-first simulations tended to favor a topology in which Bukida and Malaita were sister for 82% of parameter sets and Makira-as-sister patterns dominated with mean dispersal values above 15 km (Makira was inferred to be sister in 35.3% of Bukida-first simulations with dispersal values below 15 km and 12.9% of simulations with dispersal values above 15 km). This tendency for Bukida and Malaita to be sister, with Makira sister to that clade, occurred regardless of the colonization order among the islands: even when Makira was simulated to be colonized first and the “true” topology had Bukida and New Georgia as sister, 83% of topologies favoring Bukida and Malaita as sister with dispersal values above 15 km (not significantly different from Bukida-first simulations; Fisher’s exact test *P* = 0.83). As such, although we posit gene flow is likely biasing our empirical topology, the topology we recover with the empirical data is different from what we would have predicted based on the simulations alone. However, it is worth nothing that the sister relationship between Malaita and Bukida did not find strong support in our empirical analyses. The levels of migration that we consider in our simulations are plausible between allospecies across both island and non-island systems, suggesting interpretations of topologies that infer the most isolated taxon to be sister to all others should be interpreted with caution.

### Biogeography of Symposiachrus

Our phylogenetic analyses further elucidated the likely colonization history of the *Symposiachrus barbatus* complex. The close relationship between the recently split *S. melanopterus* from the Louisiade and D’Entrecasteaux archipelagos southeast of New Guinea (McCullough et al. 2021) and the *S. barbatus* complex puts into question which island *Symposiachrus* colonized first. Our colonization simulations showed that despite the closest point between the Solomon Islands and the range of *S. melanopterus* being the Louisiade Archipelago and the New Georgia Group, most simulations resulted in the Bukida Group being colonized first, likely due to its proximity to the larger D’Entrecasteaux archipelago.

When understanding the biogeographic history of the archipelago, it is worth nothing that the Solomon Islands are a geologically complex archipelago, and the New Georgia Group in particular saw notable uplift in the late Pleistocene (Mann et al. 1998; Cowley et al. 2004). However, land was present when *Symposiachrus* likely colonized the Solomons (1–1.4 million years ago; McCullough et al. 2022). This mid-Pleistocene colonization time implies that post-colonization variation in bathymetry (i.e., uplift and subsidence) was minimal relative to the changes induced by variable see level throughout the Pleistocene. The geographic complexity due to uplift and subsidence is not of concern for the study at hand but is an essential consideration for studies in geologically active archipelagos (Whittaker et al. 2008).

### Gene Flow and Archipelago Geography

We found evidence for gene flow within and between major island groups. Using the approach outlined in the *Estimation of Inter-Island Migration* section, we can approximate the relative probability of migration between pairs of islands (35 km mean dispersal distance used here; as in Fig. 5A). This line of inference clarifies why gene flow was inferred between the Bukida Group and Malaita but not Makira, because a prospective colonist has a 16.4x better chance of reaching Malaita from Ngella than Makira from Guadalcanal (Fig. 5A). Similarly, a migrant from Malaita has a 3.75x higher chance of reaching Makira than one from Guadalcanal to Makira.

Despite the relative proximity and favorable arrangement for gene flow (i.e., running parallel to each other) of the Bukida and New Georgia groups, the inferred admixture fraction between islands of the New Georgia and Bukida groups is, on average, negligibly different than that between Malaita and Makira (Supplementary Fig. S4). They were also not significant after correcting for multiple comparisons (although 14% of tested edges were significant at a pre-correction α = 0.05). However, this is likely driven by differences in power between different tests for gene flow, with Bukida–New Georgia comparisons included populations that experience gene flow, especially at the last glacial maximum, reducing the test’s power and likely underestimating f_4_ statistics (Durand et al. 2011). Additionally, the inference of gene flow between Malaita and Makira is consistent with the relative isolation between Makira and the Bukida group that is essential for producing the biased pattern (Fig. 5C). Indeed, at higher dispersal distances in our simulations, the moderate gene flow between Makira and Malaita resulted in them being inferred as sister (Fig. 5C).

Although this gene flow could only reflect historical processes (i.e., higher dispersal soon after colonization, DeRaad et al. 2024), our inference of an F1 intergrade on Ranongga (Fig. 3D) demonstrates an ongoing capacity for crossing modern water gaps comparable with those seen between island groups (Ranongga**–**Kolombangara 37 km, Ngella–Malaita 42 km). This inter-island intergrade is an important demonstration of the feasibility of gene flow in late stages of colonization in sedentary island birds, something that has rarely been demonstrated outside of the well-studied Darwin’s finches (Lamichhaney et al. 2018; Grant and Grant 2020).

### Impact of Over-Water Gene Flow on Phylogeographic Inference

One practical implication of these simulated and empirical data is the potential for phylogenetic inferences at certain geographic scales to reflect gene flow rather than colonization history. We interpret our finding of Makira-sister topologies in concatenated analyses as consistent with a scenario in which the isolation of Makira from the Bukida Group shapes gene flow, potentially biasing or obscuring phylogenetic inference. At the same time, we note that the lack of resolution recovered by our methods may, in our view, more accurately reflect the true underlying uncertainty in the system. At deeper time scales, such branching patterns of what were once radiations may reflect the relative time of cessation of gene flow (possibly via completion of speciation), as opposed to biogeographic patterns. Indeed, our simulations show that only the strongest branching patterns with the least gene flow correctly recover the topology for both Makira-first (Supplementary Fig. S5B) and Makira-last (Fig. 5C) colonization histories. This can be seen as an extension of the similarities between intermittently allopatric populations that experience high levels of gene flow (Smith and Filardi 2007). The Bukida Group also includes Guadalcanal, which has never been fully connected to other islands but remains poorly differentiated from other lineages (Figs. 2B and 3B). When scaled up across larger water gaps, this same logic—where inter-island gene flow drives low-level population structure—can lead to biased phylogenetic inference, even at the level of allospecies (Fig. 5C). This lack of distinguishability of alternate colonization histories with phylogenomic data due to gene flow can be viewed in parallel to the lack of distinguishability of alternate diversification histories for the same topology due to extinction (Sanmartín and Meseguer 2016; Louca and Pennell 2020). This may be further exacerbated in remote archipelagos, where extinction due to evolutionary processes and human impact is especially prominent (Wilson 1961; Steadman 1995; Wright et al. 2016).

Although non-linear colonization can occur (as we found for around 1% of our spatial simulations; Fig. 5A), the occurrence of concordant improbable topologies in multiple co-distributed taxa would support our gene flow-driven hypothesis. Indeed, there are several such lineages on Makira that are unexpectedly inferred as sister to other lineages in the archipelago. Such examples include *Ceyx* kingfishers (DeRaad et al. 2024), *Rhipidura* fantails (Klicka et al. 2022), and *Nesonycteris* blossom bats (Lavery et al. 2023). If colonization followed by gene flow predominates, it holds that the most difficult to access location in a focal region would also be inferred as sister to other taxa in that clade, because the number of migrants relates both to colonization and gene flow (MacArthur and Wilson 1967; Gyllenhaal et al. 2020).

### Relation to Island Diversification

Several models of island evolution emphasize a decrease in dispersal ability over time (Wilson 1961; Moyle et al. 2009; Wright et al. 2016), which DeRaad et al. (2024) note can resolve the “paradox” of taxa differentiating across water gaps much narrower than they had to cross to colonize many islands of an archipelago. This model of evolution, in which dispersal ability starts high and decreases over time, should have an impact on phylogenetic inference in the same way we have outlined here, with topologies impacted by the relative isolation of populations during their divergence. If gene flow ceases, either due to reduced dispersal or the onset of reproductive isolation, phylogenetic topology can preserve a historical signal of geographic proximity rather than colonization history. Conversely, if dispersal ability increases—reinitiating the expansion phase of the taxon cycle—lineages may colonize new archipelagos. Indeed, intra-archipelago speciation accounts for most known cases of secondary sympatry in the South Pacific (e.g., Cibois et al. 2014; Andersen, Shult, et al. 2015, Cowles and Uy 2019; Manthey et al. 2020; Brady et al. 2022; Shogren et al. 2024). The only clearly documented case of completed speciation within a Solomons-endemic clade of birds may be the *Phylloscopus* warblers on Kolombangara, which exhibit strong ecological divergence and a phylogeny heavily influenced by gene flow (DeCicco et al. 2024). However, the completion of speciation through attainment of secondary sympatry can only be observed if contact is attained. The distinctive *Symposiachrus vidua* of Makira, for example, may be a biological species that simply lacks the dispersal ability to achieve secondary sympatry. It has evolved divergent traits, including a scaled wing pattern and lack of white in the cheek (comparable with plumage divergence in *Monarcha* inferred to be associated with assortative mating by Uy, Moyle, Filardi 2009; Uy, Moyle, Filardi, Cheviron 2009), and unique foraging strategies and interspecific interactions (Weeks et al. 2020). Future simulation studies could clarify how variation in dispersal ability throughout the divergence process shapes the impact of gene flow on phylogenetic inference.

## Supplementary Material

syaf081_Supplemental_Files

## Data Availability

Analysis scripts and phylogenetic and population genetic input files are uploaded to Dryad (https://doi.org/10.5061/dryad.15dv41p02). Raw Illumina sequencing reads for RAD-seq and new UCE data are available from the NCBI SRA (BioProject PRJNA1254294). Analysis scripts and some input files will also be uploaded to GitHub (https://github.com/ethangyllenhaal/SolomonsSymposRad).

## Supplementary Material

Data available from the Dryad Digital Repository: https://doi.org/10.5061/dryad.15dv41p02
