# Supplementary material for: Gene Flow Complicates Phylogenetic Inference in an Archipelago Radiation
Source: Syst Biol. 2025 Nov 12;75(4):694–711. doi: 10.1093/sysbio/syaf081 (PMC13260659; doi:10.1093/sysbio/syaf081)
Supplement: syaf081_Supplemental_Files [file syaf081_supplemental_files.zip › Gyllenhaal_SoloSympos_Revision2_Appendix_SupplementalMaterial.docx]

Supplementary Text

Assessing a putative intergrade

Included in our sampling was one bird from Ranongga Island (typically *S. browni ganongae*) that had an intermediate phenotype, leading us to hypothesize that it was a first-generation immigrant or intergrade. We included this individual to test if it represented a modern case of overwater migration.

To evaluate this unusually plumaged bird, we generated input with PLINK v1.90b6.21 (Chang et al. 2015) to perform a model-based clustering for New Georgia Group individuals using the program ADMIXTURE v1.3.0 (Alexander & Lange 2011). We confirmed the hybrid status of the unusual bird by calculating heterozygosity at sites fixed between our samples of the two subgroups of the New Georgia group (Ranongga and Vella compared to all other island in the group), following the general approach of *introgress*; Gompert & Buerkle 2010.

Detailed Colonization Simulation

We generated raster files in PleistoDist v1.1.0 (Tan et al. 2022) for modern sea levels Bintanja & van de Wal 2008) and converted them to PNG files using ImageMagick v6.9.7-4. The output PNG file was further modified to reflect the extant range of the sister taxon, i.e., only including the Louisiade and D’Entrecasteaux archipelagos as sources. The modified file was used to make a map for spatially explicit non-Wright-Fisher SLiM 4 simulations. We followed an approach to simulating dispersal first outlined in Tan et al. (2022), allowing for offspring to engage in short-distance over-land dispersal near the parent and long-distance dispersal that could cross large distances (i.e., water gaps). It emulates the same equation in Fig. 1b, but in a geographically explicit framework where area, target size, and inter-island distance arise naturally from the map. The long-distance dispersal had a 0.5% probability of occurring (corresponding to α in Fig. 1b) and was simulated by randomly choosing a movement direction and maximum distance. The maximum distance was randomly generated using an exponential dispersal kernel (Fig. 1b) and was repeated for a range of mean dispersal values (a wider range than what MacArthur & Wilson 1967, 1967 considered for birds, and that Gyllenhaal et. al. 2020 used for testing empirical estimates). Then, a dispersing individual’s position was checked every 10 pixels until it first crossed water. Once over water, its position was checked again every 10 pixels until land was encountered, after which its new position was changed to the first point it landed. If it did not reach land, the individual was removed from the simulation. Because we modeled this as a continuous space, we checked the spatial position of individuals to assign them to island groups. We used SLiM’s tag functionality to mark individuals as coming from a given island. We checked every 10 generations whether islands were occupied by >50 individuals and recorded the first generation in which it attained a population of >50, in addition to the mean tag value to assess the source of colonization. We then estimated colonization order and resulting topologies based on the tag values of colonized islands. We ran 100 replicates in GNU Parallel v202109022 (Tange 2021) for a range of mean long-distance dispersal values (200, 400, 600, 800, 1000, 1200, and 1600 pixels in the map, where 10 pixels = approximately 4.5 km). Simulations were run for 30,000 generations.

Supplementary Figures

Figure S1: Principal component plots of genomic data for the New Georgia group, with labels corresponding to island names (with the exception of the intergrade, Figure 3D). A) A map of the New Georgia group with colors corresponding to populations in the PCAs. B) First and second principal component for all New Georgia Group samples. C) Second and third principal component for all New Georgia samples, to illustrate the separation of Vella Lavella and Ranongga in the third principal component.


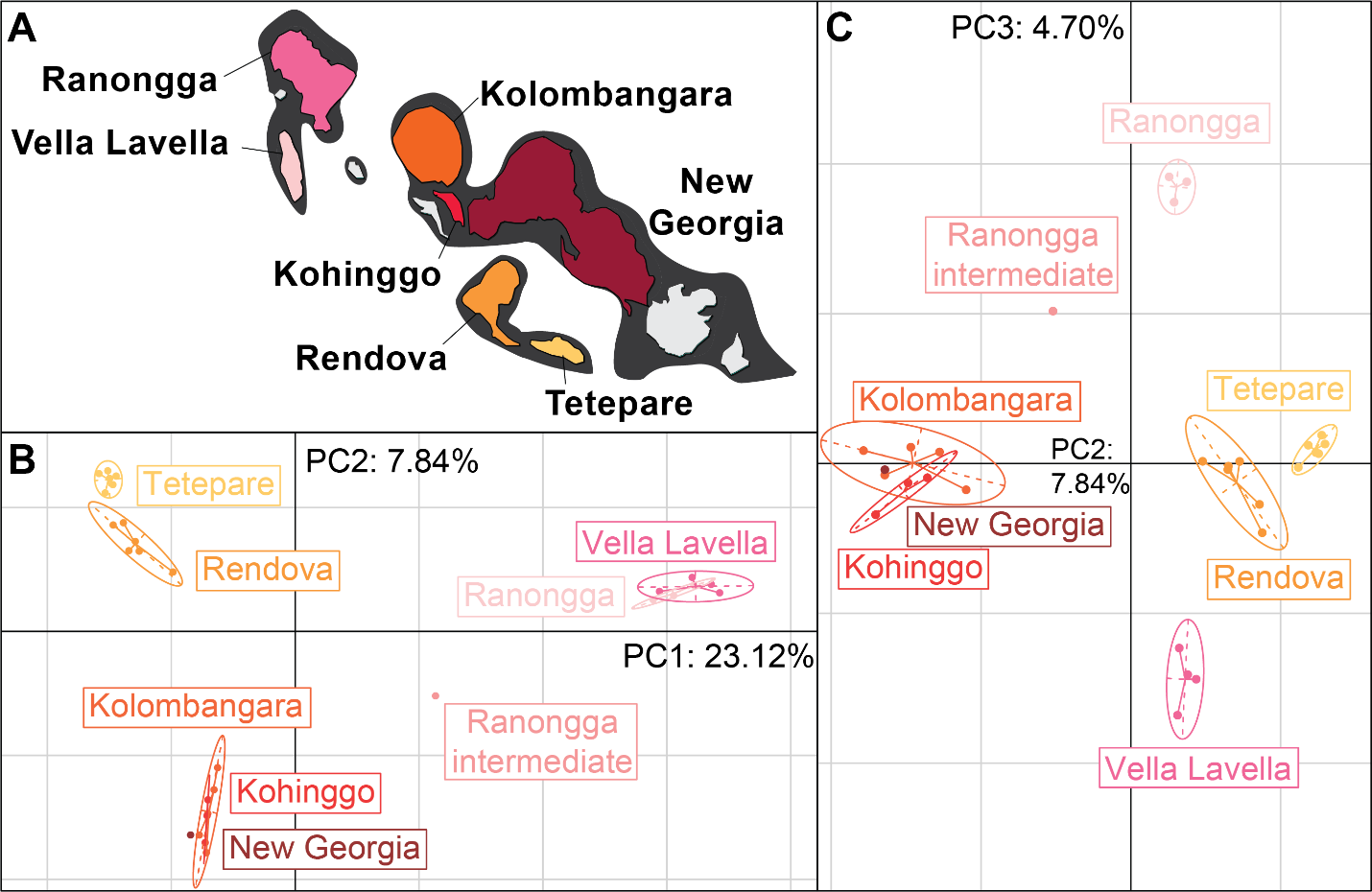


Figure S2: A) Map of the Solomon Islands with island name abbreviations associated with tip labels of B) a maximum likelihood phylogeny generated in IQTREE of all RAD-seq samples that passed filtering. Labels of the phylogeny also include the specimen number associated with Table S1. Geographic notation was omitted for outgroups.


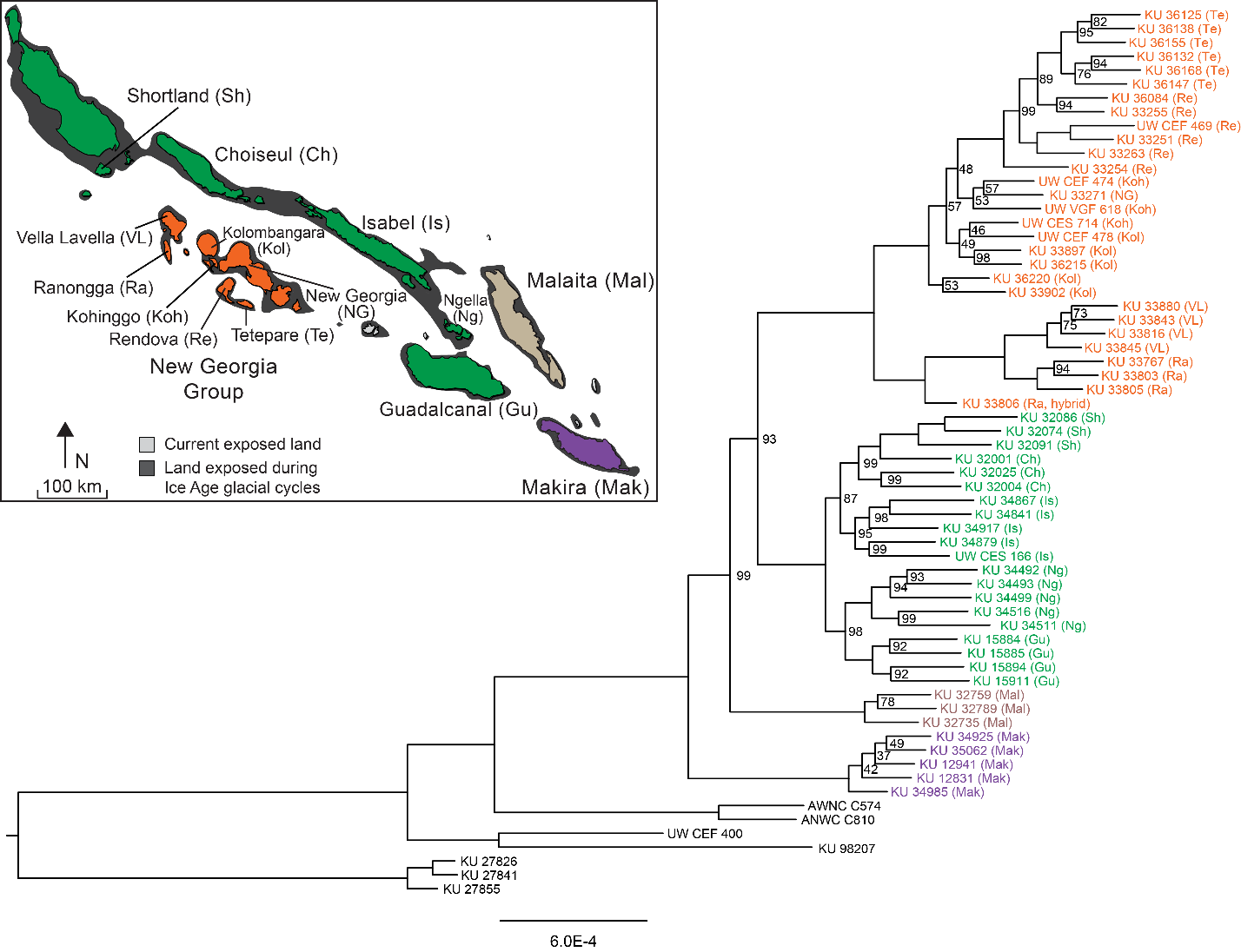


Figure S3: A) Map of the Solomon Islands colored according to island group and labeled with abbreviations of island names used in later subfigures. B) SNAPP tree with 2 random samples from select New Georgia and Bukida group islands (two islands per group), 4 random samples from Makira, all 3 Malaita samples, and 2 outgroup samples (*S. trivirgatus*). C) SNAPP tree with 2 random samples from most New Georgia and Bukida group islands (five islands per group), all 5 Makira samples, all 3 Malaita samples, and 2 outgroup samples (*S. trivirgatus*). D) Summary of trees in posterior of SNAPP analysis generated by TreeAnnotator for datasets with i) 3 samples from a single island per island group (as in Figure 4D), ii) two islands for multi-island groups (see B), and iii) five islands per multi-island group (see C).


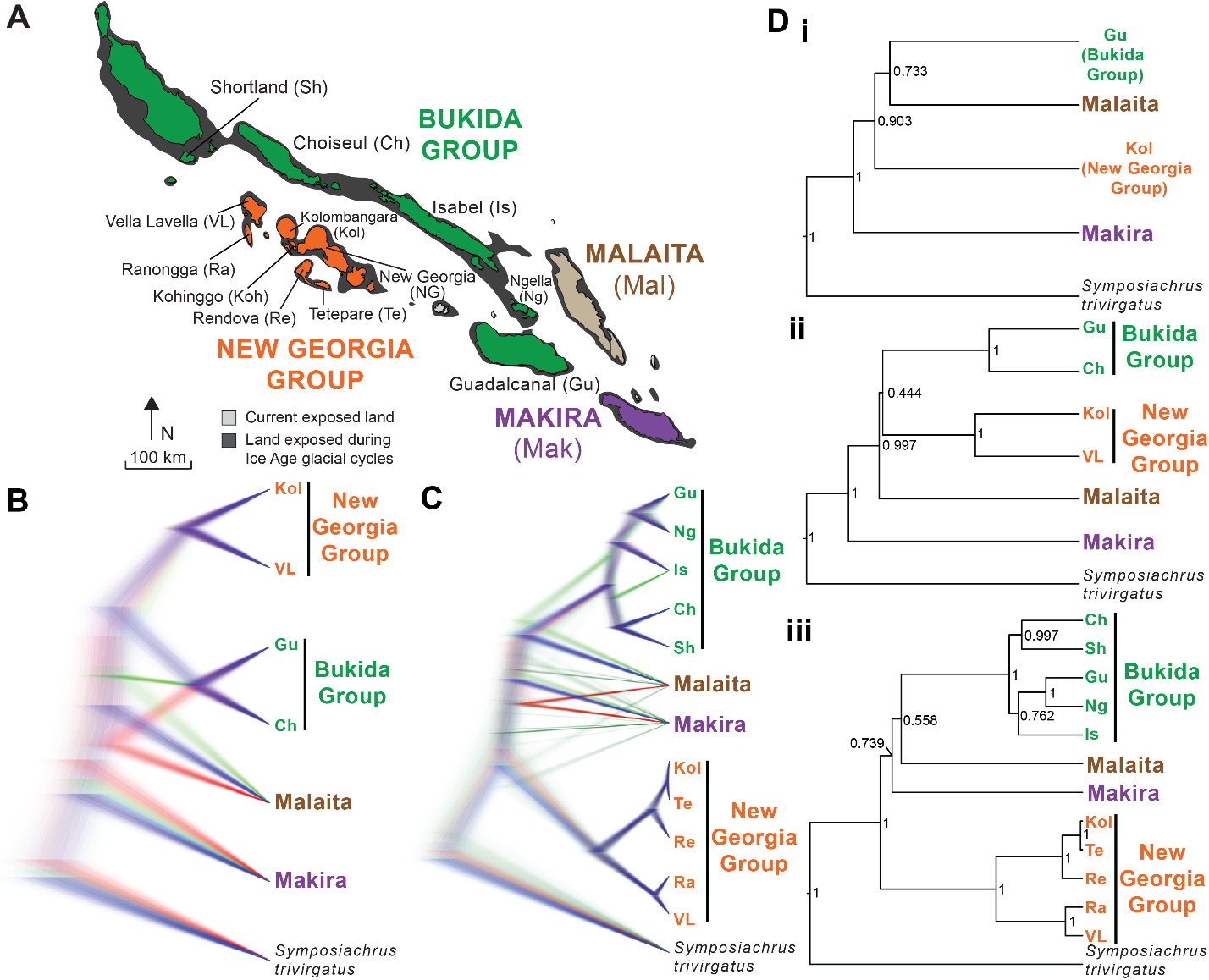


Figure S4: Figures representing significant values of the f_branch_ statistic, with the darkness of the colored squares representing the inferred value. Gray squares represent ones that could not be tested for gene flow. Axes have phylogenies that were tested, with dashed lines representing gene flow with ancestral nodes, with text color at the tips corresponding to A) the map of the Solomon Islands (colors correspond to island group, not individual islands). Topologies tested were B) the RAxML topology with Malaita included, C) the RAxML topology without Malaita, D) a conservative topology with certain populations removed to avoid phylogenetic uncertainty, E) the conservative topology with Malaita removed, F) a topology excluding the New Georgia Group, and G) a topology excluding the New Georgia Group and Isabel. Downsampling was done to H) three and I) two individuals, using the topology in B. Subfigures C–I demonstrate that the major findings depicted in Figure 5 are robust to topological uncertainty and downsampling.


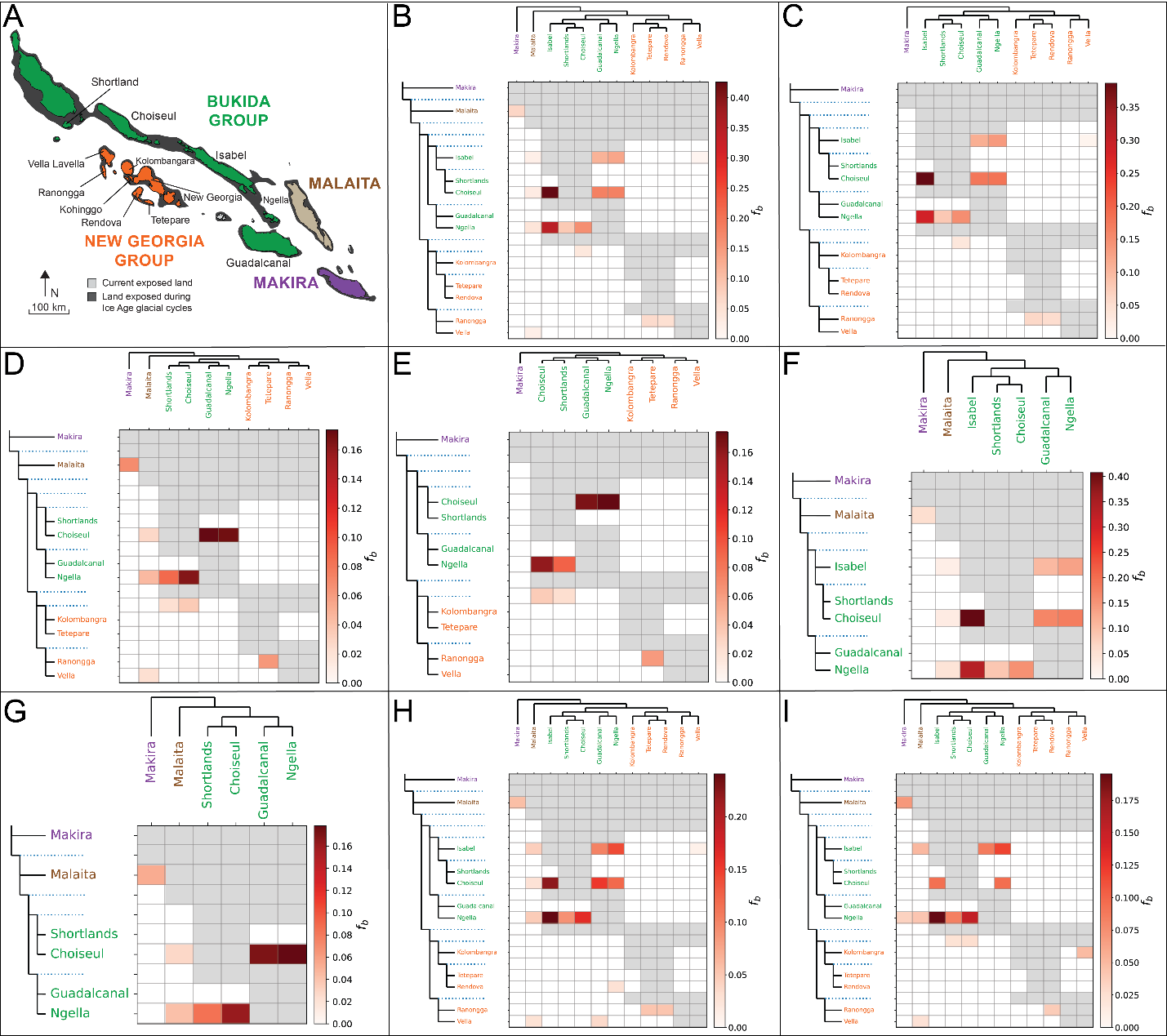


Figure S5: Summary of results from our full set of coalescent simulations, both for a) simulations starting in the Bukida group and b) simulations starting in Makira (with different true topologies). The color of each square corresponds to the most frequent topology, and transparency corresponds to the proportion of simulations that recovered the true topology in B. For visualization purposes, the dominant topology was first assigned as unresolved if less than 50% of the trees found a well-supported topology of monophyletic island groups, and if not, the dominant topology represents the majority of the remaining, well-resolved topologies. The inner axis labels correspond to variation in effective population size and dispersal within each set of tiles, while the outer labels correspond to values of split times that are fixed for a given set of tiles.


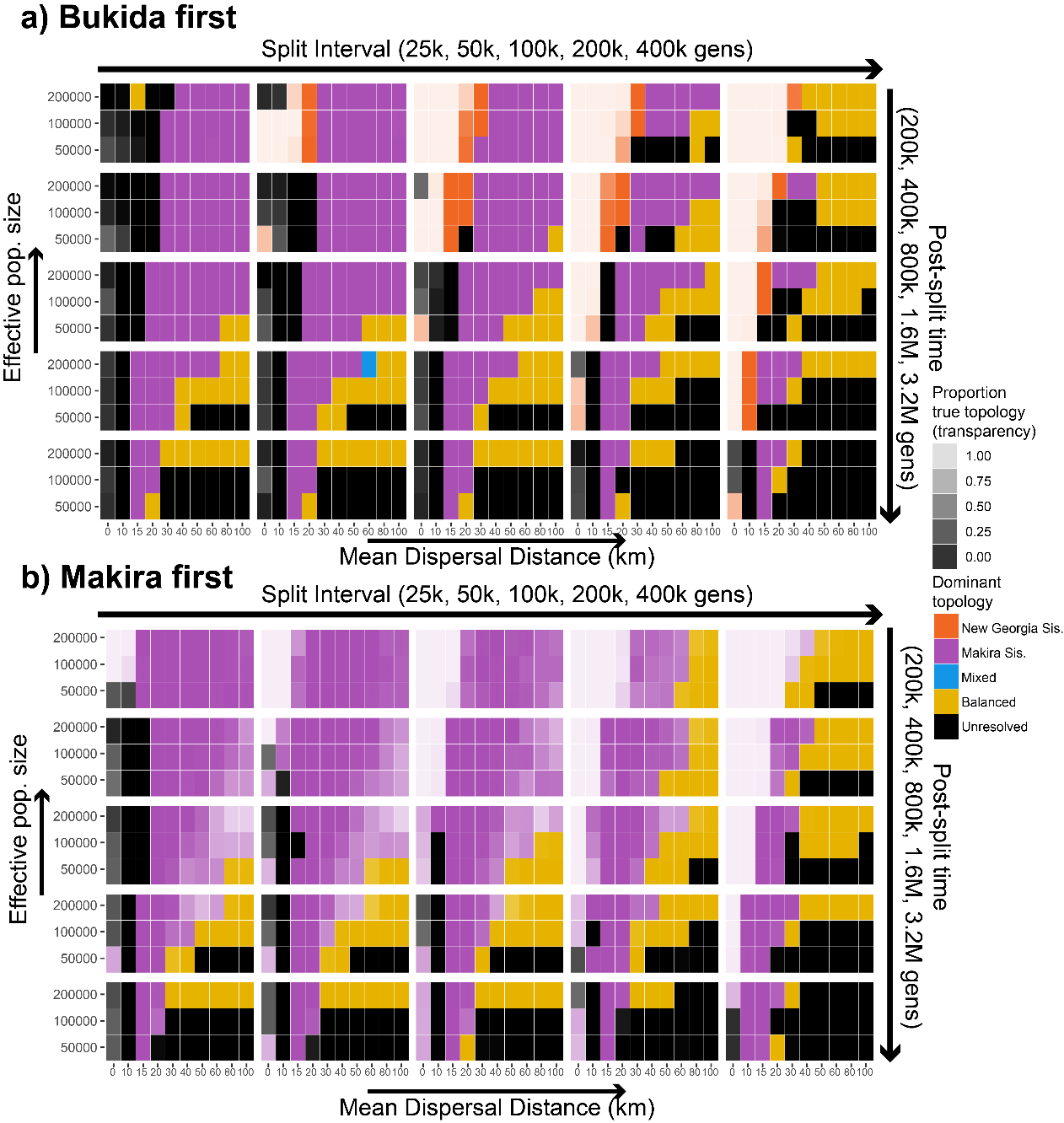


Table S1: Table of samples used for RAD-seq dataset. From left to right, columns are species name, subspecies (when relevant), island, country, voucher number, tissue number, sample name used in scripts, sample name used for SRA accession, number of reads, which datasets the sample was included in, and Arctos database links. The numbers in the dataset column correspond to: 1) full dataset, 2) all Solomons samples, 3) all New Georgia samples, 4) all Bukida samples, 5) samples used in DSuite tests for gene flow, and 6) samples used in SNAPP. **[IN DRYAD UPLOAD]**

Table S2: Table of samples used for UCE dataset. From left to right, columns are species name, subspecies (when relevant), island, country, voucher number, tissue number (when relevant), sample name used in scripts, sample name used for SRA accession, and number of recovered loci. **[IN DRYAD UPLOAD]**

Table S3: Table of pairwise FST values between islands. Island groups (i.e., New Georgia and Bukida) are demarcated by thicker lines. Comparable cells above and below the diagonal are the same value. Abbreviations are as follows: Sho, Shortland; Cho, Choiseul; Isa, Isabel; Nge, Ngella; Gua, Guadalcanal; Mal, Malaita; Koh, Kohinggo; Kol, Kolombangara; Ren, Rendova; Tet, Tetepare; Ran, Ranongga; Vel, Vella Lavella; Mak, Makira.

| Pop. | Sho | Cho | Isa | Nge | Gua | Mal | Koh | Kol | Ren | Tet | Ran | Vel | Mak |
| --- | --- | --- | --- | --- | --- | --- | --- | --- | --- | --- | --- | --- | --- |
| Sho | - | 0.082 | 0.113 | 0.171 | 0.174 | 0.356 | 0.355 | 0.360 | 0.382 | 0.439 | 0.445 | 0.463 | 0.437 |
| Cho | 0.082 | - | 0.036 | 0.097 | 0.093 | 0.300 | 0.311 | 0.323 | 0.345 | 0.405 | 0.405 | 0.426 | 0.404 |
| Isa | 0.113 | 0.036 | - | 0.071 | 0.074 | 0.269 | 0.284 | 0.296 | 0.324 | 0.371 | 0.364 | 0.381 | 0.371 |
| Nge | 0.171 | 0.097 | 0.071 | - | 0.075 | 0.294 | 0.313 | 0.323 | 0.348 | 0.397 | 0.396 | 0.412 | 0.394 |
| Gua | 0.174 | 0.093 | 0.074 | 0.075 | - | 0.293 | 0.307 | 0.316 | 0.340 | 0.396 | 0.398 | 0.412 | 0.396 |
| Mal | 0.356 | 0.300 | 0.269 | 0.294 | 0.293 | - | 0.397 | 0.397 | 0.409 | 0.469 | 0.484 | 0.495 | 0.456 |
| Koh | 0.355 | 0.311 | 0.284 | 0.313 | 0.307 | 0.397 | - | 0.015 | 0.087 | 0.149 | 0.324 | 0.348 | 0.472 |
| Kol | 0.360 | 0.323 | 0.296 | 0.323 | 0.316 | 0.397 | 0.015 | - | 0.099 | 0.148 | 0.313 | 0.336 | 0.462 |
| Ren | 0.382 | 0.345 | 0.324 | 0.348 | 0.340 | 0.409 | 0.087 | 0.099 | - | 0.049 | 0.334 | 0.358 | 0.474 |
| Tet | 0.439 | 0.405 | 0.371 | 0.397 | 0.396 | 0.469 | 0.149 | 0.148 | 0.049 | - | 0.400 | 0.423 | 0.522 |
| Ran | 0.445 | 0.405 | 0.364 | 0.396 | 0.398 | 0.484 | 0.324 | 0.313 | 0.334 | 0.400 | - | 0.151 | 0.537 |
| Vel | 0.463 | 0.426 | 0.381 | 0.412 | 0.412 | 0.495 | 0.348 | 0.336 | 0.358 | 0.423 | 0.151 | - | 0.544 |
| Mak | 0.437 | 0.404 | 0.371 | 0.394 | 0.396 | 0.456 | 0.472 | 0.462 | 0.474 | 0.522 | 0.537 | 0.544 | - |

Table S4: Summary of gene trees metrics with at least 2, 1, and 3 parsimony-informative sites (2 is first to match focus in paper). For each sub-table, the letters B, N, L, and K refer to the sample from the Bukida, New Georgia, Malaita, and Makira groups. The “proportion” refers to the proportion of the gene trees supporting a given pattern, with “X sister” referring to topologies where the other 3 groups form a clade to the exclusion of X population. The “mean distance” is the mean branch length from the “sister” group to the other 3 groups (or average distance between non-sister pairs for the balanced topology), and the within-clade distance is the mean branch length between members of the rest of the tree (or the mean of each sister pair for the balanced topology).

| **2 Parsimony-informative Sites** | | | | | | | | |
| --- | --- | --- | --- | --- | --- | --- | --- | --- |
|  | B sister | N sister | L sister | K sister | B-N balanced | Other | Total trees |  |
| Proportion | 0.1741 | 0.1830 | 0.1161 | 0.2768 | 0.1116 | 0.1384 | 224 |  |
| Mean distance | 0.0499 | 0.0356 | 0.0434 | 0.0264 | 0.0480 |  |  |  |
| Within-clade | 0.0110 | 0.0105 | 0.0082 | 0.0165 | 0.0019 |  |  |  |
| **1 Parsimony-informative Sites** | | | | | | | | |
|  | B sister | N sister | L sister | K sister | B-N balanced | Other | Total trees |  |
| Proportion | 0.1913 | 0.2323 | 0.1240 | 0.3202 | 0.0501 | 0.0821 | 1218 |  |
| Mean distance | 0.0202 | 0.0150 | 0.0198 | 0.0138 | 0.0204 |  |  |  |
| Within-clade | 0.0080 | 0.0062 | 0.0058 | 0.0094 | 0.0017 |  |  |  |
| **3 Parsimony-informative Sites** | | | | | | | | |
|  | B sister | N sister | L sister | K sister | B-N balanced | Other | Total trees |  |
| Proportion | 0.1930 | 0.1754 | 0.1404 | 0.2632 | 0.0702 | 0.1579 | 57 |  |
| Mean distance | 0.0396 | 0.0464 | 0.0857 | 0.0280 | 0.1560 |  |  |  |
| Within-clade | 0.0100 | 0.0021 | 0.0107 | 0.0218 | 0.0051 |  |  |  |

Table S5: Table summarizing tests for multiple comparisons in ABBA/BABA tests, per category of edge. Columns are the category of gene flow edge tested, the number of tests, the harmonic mean p-value for the category, if the category had significant tests before correction and after correction, and a list of comparisons in newick notation as ((P1,P2),P3).

| Category | Tests | Harmonic p-value | Significant before B-H? | Significant after B-H? | Comparisons significant after B-H correction |
| --- | --- | --- | --- | --- | --- |
| Within Bukida | 10 | 0.00002 | Yes | Yes | ((Guadalcanal, Ngella), Choiseul) ((Shortland, Choiseul), Guadalcanal) ((Shortland, Isabel), Guadalcanal) ((Shortland, Choiseul), Isabel) ((Guadalcanal, Ngella), Isabel) ((Shortland, Choiseul), Ngella) ((Ngella, Isabel), Shortland) ((Guadalcanal, Ngella), Shortland) |
| Bukida-Malaita | 10 | 0.00655 | Yes | Yes | ((Shortland, Isabel), Malaita) ((Guadalcanal, Ngella), Malaita) ((Shortland, Ngella), Malaita) |
| Malaita-Makira | 10 | 0.00951 | Yes | Yes | ((Choiseul, Malaita), Makira) ((Guadalcanal, Malaita), Makira) ((Isabel, Malaita), Makira) ((Kolombangra, Malaita), Makira) ((Ngella, Malaita), Makira) ((Ranongga, Malaita), Makira) ((Rendova, Malaita), Makira) ((Tetepare, Malaita), Makira) ((Vella, Malaita), Makira) |
| Within New Georgia | 10 | 0.02364 | Yes | Yes | ((Vella, Ranongga), Tetepare) |
| Bukida-New Georgia | 113 | 0.05882 | Yes | No |  |
| New Georgia-Malaita | 22 | 0.19222 | Yes | No |  |
| Bukida-Makira | 34 | 0.19583 | No | No |  |
| New Georgia-Makira | 11 | 0.28308 | No | No |  |

Table S6: Full table of colonization simulation results, summarized in Figure 5A. The first column represents the mean dispersal distance in pixels (10 pixels corresponds to approximately 4.5 km). The second column corresponds to the number of simulations where enough islands were colonized to assess the full order. The rest of the columns correspond to the order islands were colonized. For example, NKBL means that New Georgia (N), Makira (K), Bukida (B), and Malaita (L) were colonized in that order. Source populations were always from the original source or adjacent islands, and often mixed. Cell values correspond with the number of successful simulations where a colonization order was found (zero values were removed).

| Dispersal | Finished | NKBL | NBLK | NBKL | LBKN | KNBL | KLBN | KBNL | BNLK | BNKL | BLNK | BLKN |
| --- | --- | --- | --- | --- | --- | --- | --- | --- | --- | --- | --- | --- |
| 200 | 0 | 0 | 0 | 0 | 0 | 0 | 0 | 0 | 0 | 0 | 0 | 0 |
| 400 | 24 | 0 | 8 | 0 | 0 | 0 | 0 | 2 | 13 | 0 | 1 | 0 |
| 600 | 125 | 0 | 37 | 0 | 0 | 0 | 0 | 0 | 83 | 1 | 1 | 3 |
| 800 | 184 | 0 | 50 | 1 | 1 | 0 | 0 | 1 | 123 | 4 | 1 | 3 |
| 1000 | 200 | 0 | 59 | 1 | 0 | 1 | 1 | 0 | 130 | 1 | 4 | 3 |
| 1200 | 200 | 0 | 66 | 2 | 0 | 0 | 1 | 0 | 121 | 3 | 2 | 5 |
| 1600 | 197 | 1 | 47 | 1 | 0 | 1 | 0 | 0 | 140 | 2 | 1 | 4 |
| Total | 930 | 1 | 267 | 5 | 1 | 2 | 2 | 3 | 610 | 11 | 10 | 18 |
